# The Survival of the Fittest

**Published:** 1878-02

**Authors:** 


### Editorial.

### THE SURVIVAL OF THE FITTEST.

That there are more physicians to each one thousand of inhabitants in this country than in any other, is a fact which is challenging the attention of those not specially interested in statistics. And the end is not yet. There is no immediate prospect of a decrease in either the number or capacity of the institutions from which the ranks of the profession are recruited. On the contrary, since the causes which have operated to produce the fecundity of these institutions are still active, we may even look for their further development in numbers and capacity. A constantly increasing stream of young graduates flows from the door of each, eager to test for themselves the question whether there is room for them in the professional world.

The radical differences between these institutions are known to all medical men. Some of them we recognize as qualified to properly educate in science; many are unquestionably not thus qualified. The latter class comprises not only several of the colleges labelled "regular," but also those entitled "homeopathic," "eclectic," "physio-medical," "physio-eclectic-medical," "hygienic," and whatever other names avarice and folly may have suggested, in order to disseminate delusion, or, what is even worse than delusion, a system of half-truths.

Nor is this all. The particular branch of American industry to which we refer, has never been protected from competition with the "pauper labor of Europe," by the imposition of a discriminating or restrictive tariff. To these shores the man of foreign medical education and birth is free to come, here to engage in practice side by side with his home-made professional brother, and to still further diminish the assuredly limited fund upon which they must all depend for sustenance. Canada, England, Ireland, Germany, France, Sweden and Norway have medical representatives in most of our towns and country places, here and there, in really large numbers, who hope to receive support from those of their own nationality, and, if possible, to secure a still wider patronage.

The causes operating to produce this undue multiplicity of physicians in America do not require discussion, they are too well understood. It is true that, of late, a widespread financial depression has choked the avenues of trade, made commercial enterprises unprofitable, and thus contributed to crowding the benches of medical schools with professional students. But there is a cause of greater significance. It lies in the fact that the vast majority of medical schools in the United States are destitute of a proper foundation, as well as of national and State support. Had they depended for their sustentation upon such sources, self-interest would have impelled their various Faculties to maintain such a standard as would have discouraged an excessive number of applicants for their honors. But self-interest has operated in a precisely opposite direction. The schools depend for their support upon the fees which are paid to professors, a class of men actively engaged in professional pursuits. The income of each school has been proportioned to the number of its graduates, and in some instances the remuneration has been large. The schools have been thus directly interested not so much in the task of elevating the standard of medicine as in securing the largest number of names on the roll of students, and a correspondingly large financial exhibit.

The stream can rise no higner than its source. The mass of medical men in the country have become such on leaving the portals of these same institutions, and naturally entertain for them the somewhat tender regard of an alumnus for his alma mater. Though they greatly outnumber those who are engaged in the task of still further crowding their ranks, it does not seem to have occurred to them to exert, as a body, any restraining influence upon their too fertile mothers, by insisting that they themselves should have a voice in deciding who should be allowed to compete with them in the struggle for a share of the family loaf. Even should they attempt this, it is doubtful if they could succeed for a moment in staying the course of each annual gestation and delivery.

All are interested in asking what the result of this is to be in the future. The immediate results are known to too many. The college professor, whose eloquence and learning have long increased the attendance upon his classes, and thus contributed to putting money in his purse, finds some of his quondam pupils settling down on either side of his door, and disputing with him quietly, gradually and yet inevitably, inch by inch, the ground which was once his own. A large number of the fledglings, it is true, early or late abandon the profession, but many remain, striving for a success which never comes, their hearts sinking as hope is long deferred. Others, the favorites of rare circumstances or fortune, sorrowfully regard those who are gradually forced downward, till they drink the dregs of dissipation and debt. Some become the shabby-genteel dependents upon the resources of their friends. The fate of a smaller number is indeed wretched. A few years ago, a well-educated and highly intelligent young physician in New York city, who had taken a special degree in Vienna, blew out his brains in despair over his remediless condition in life. These are some of the results. They who achieve a great success (and many, we are pleased to say it, do this), simply tread great obstacles under foot.

We use the present tense advisedly, for the big bonanzas of practice have long been pre-empted, and the royal roads to honor and wealth in the profession are crowded thoroughfares, where wearied toilers jostle each other by the way. The time is forever passed when the half-educated professional man in this country can become eminent from the force of exceptionally favorable circumstances or the absence of keen competition.

What, we repeat, will be the outcome of all this? If the answer to that question was impressed deeply upon the minds of the students and professional men of this country, it would be well for all.

When the operation of natural laws is untrammeled, the result is always beneficial to mankind, even though one class suffer.

The selfishness of the human family is the key-stone of commerce and civilization, and the selfish instinct of the corporations we call medical schools will in the end produce a body of medical men in this country that have never been equalled, and never could have been graduated from universities depending upon endowments. A desperate struggle for existence will soon, if it does not already, await each candidate for professional success, and in that struggle only the fittest will survive. He who can best cut for stone, treat a fever or deliver a woman in perilous travail, will surpass him who cannot do these things as well. And, again, in every community, of those capable of doing these things equally well, he who has the broadest education, the best trained intellect and the most correct judgment will bear the palm. It is said that "blood will tell," and so, indeed, in the long run, will education, training, skill, hospital experience, and fertility of resources in emergencies. They will tell in the struggle for professional existence. Let no man suffer himself to become blind to his deficiencies in these matters.

It has been said that the greatest men the world has ever seen only surpassed by a little the great men around them. Italy had never produced so many masters in art as in the day when Raphael portrayed in glowing colors his wonderful conceptions, and it is probable that Italy never saw so many indifferent and inferior works of art as in that day. In what numberless directions was American ingenuity vainly reaching when two men, each ignorant of the other's idea, simultaneously invented the sewing machine!

When physicians constitute one per cent. of the adult population of this country (and they will soon be represented in that ratio, if the present rate of increase be maintained), the great among them will be great indeed. The standards of the old world will then be rapidly reached and surpassed. There never was another hour when medicine attracted as much attention in this country. At the head of its various departments stand eminent Americans, whose position was never so fully recognized abroad as at present. There are also probably more half educated medical men in the land than ever before in this or any other. American medical literature was never so abundant as now, nor have its best representatives been ever so valuable and its poorest so worthless.

The difference between the red-streaked pole of the barber-surgeon of old England and the title of the Fellow of the Royal College to-day, is a measure of the distance between the lowest and the highest of us; between the physicians of the present and the future. The process of natural selection is evidently to continue till, in the struggle for existence, the weakest go to the wall and the fittest survive. And of the survivors shall come kings among men.

